# Impact of IDH Mutations, the 1p/19q Co-Deletion and the G-CIMP Status on Alternative Splicing in Diffuse Gliomas

**DOI:** 10.3390/ijms24129825

**Published:** 2023-06-06

**Authors:** Lu Zhang, Sabrina Fritah, Petr V. Nazarov, Tony Kaoma, Eric Van Dyck

**Affiliations:** 1 Bioinformatics Platform, Data Integration and Analysis Unit (DIA), Luxembourg Institute of Health (LIH), L-1445 Strassen, Luxembourg; lu.zhang@lih.lu (L.Z.); petr.nazarov@lih.lu (P.V.N.); 2 NorLux Neuro-Oncology Laboratory, Department of Cancer Research (DoCR), Luxembourg Institute of Health (LIH), L-1445 Strassen, Luxembourg; sabrina.fritah@lih.lu; 3 Multiomics Data Science Research Group, DoCR, Luxembourg Institute of Health (LIH), L-1445 Strassen, Luxembourg; 4 DNA Repair and Chemoresistance Group, DoCR, Luxembourg Institute of Health (LIH), L-1445 Strassen, Luxembourg

**Keywords:** diffuse gliomas, isocitrate dehydrogenase (IDH), 1p/19q co-deletion, G-CIMP, pre-mRNA splicing, alternative splicing, differential gene expression, epigenetic regulation

## Abstract

By generating protein diversity, alternative splicing provides an important oncogenic pathway. Isocitrate dehydrogenase (*IDH*) 1 and 2 mutations and 1p/19q co-deletion have become crucial for the novel molecular classification of diffuse gliomas, which also incorporates DNA methylation profiling. In this study, we have carried out a bioinformatics analysis to examine the impact of the *IDH* mutation, as well as the 1p/19q co-deletion and the glioma CpG island methylator phenotype (G-CIMP) status on alternative splicing in a cohort of 662 diffuse gliomas from The Cancer Genome Atlas (TCGA). We identify the biological processes and molecular functions affected by alternative splicing in the various glioma subgroups and provide evidence supporting the important contribution of alternative splicing in modulating epigenetic regulation in diffuse gliomas. Targeting the genes and pathways affected by alternative splicing might provide novel therapeutic opportunities against gliomas.

## 1. Introduction

Recent advances in our understanding of the molecular alterations underlying diffuse gliomas have led to a novel classification based on molecular markers including isocitrate dehydrogenase (*IDH*) 1 and 2 mutations and 1p/19q co-deletion (codel) [1]. It is now recognized that IDH-wild-type (WT) gliomas essentially represent primary glioblastomas (GBM), while IDH-mutant gliomas bearing (1p/19q codel) or not bearing (1p/19q non-codel) the co-deletion of 1p/19q encompass tumors previously classified as oligodendrogliomas and astrocytomas, respectively. The subsequent incorporation of DNA methylation profiling, based on the discovery of a CpG island methylation phenotype (CIMP) in a subset of gliomas [2], led to a refinement in the subclassification of IDH-mutant 1p/19q non-codel gliomas in G-CIMP-high and G-CIMP-low subgroups (reviewed in [3,4]). Notably, all of the 1p/19q codel and GCIMP-high gliomas are mutated in *IDH1* or *IDH2* [2,5,6].

A recent analysis of the alternative splicing landscape of pediatric and adult high-grade glioma (HGG) has uncovered an increased splicing burden compared with that in normal brain [7]. However, how IDH mutations, the co-deletion of 1p/19q and the G-CIMP phenotype affect alternative mRNA splicing and the expression of protein isoforms in diffuse gliomas remains largely unknown.

In gliomas, IDH mutations are found primarily in *IDH1* (incidence > 70%), with R132H representing > 90% of all mutations identified. Mutations in *IDH2*, affecting the analogous residue R172, have also been identified, but they are much rarer [8,9]. Of note, IDH mutations are also found in multiple other tumors [10]. At the mechanistic level, the IDH mutations define a neomorphic activity. Specifically, whereas WT IDH catalyzes the conversion of isocitrate into α-KG, mutant IDH converts α-KG into oncometabolite 2HG, which is an inhibitor of multiple αKG-dependent dioxygenases, including DNA/RNA demethylases, histone demethylases and proline/lysine hydroxylases [10,11]. In addition to metabolic alterations [12,13,14], the accumulation of 2HG impacts the removal of crucial epigenetic marks, leading to alterations in a variety of cellular programs affecting cellular metabolism, cancer biology and oncogenesis [10,14,15]. Notably, the TET family of DNA hydroxylases involved in DNA demethylation [16] is a major pathological target of the IDH mutations [10]. Thus, neomorphic IDH1-mutant activity leads to a glioma CpG island methylator phenotype (G-CIMP) characterized by concurrent promoter hypermethylation and the silencing of a large number of genes [2,6]. As for the *IDH* mutations [17], and contrasting with our knowledge of the impact of the G-CIMP phenotype on the DNA methylome and transcriptome in gliomas [2,4,18], very little is known about the impact of G-CIMP status on alternative mRNA splicing and the expression of protein isoforms.

The list of αKG-dependent dioxygenases acting on DNA/RNA and affected by the *IDH* mutations also contains crucial erasers of a fundamental epigenetic mark affecting RNA: the ALKBH5 and FTO dioxygenases that mediate oxidative removal of N6-methyladenosine (m6A) in RNA [19,20]. This modification, which defines a dynamic RNA epigenetic code, is interpreted by specific readers that regulate a variety of biological processes, including the alternative splicing of precursor (pre)-mRNA—a process that dramatically increases the complexity of gene expression [21,22,23]. Epigenetic readers of the m6A-RNA mark involved in pre-mRNA splicing include HNRNPA2B1 [24], HNRNPC [25], YTHDC1 [26] and SRSF2 [27], whose interaction with RNA is modulated by RNA methylation. Compounding the problem, the activity of crucial splicing factors such as U2AF65 has been found to be directly regulated through protein post-translational modification (i.e., lysyl-5-hydroxylation) by the αKG-dependent lysine hydroxylase Jmjd6, with important biological consequences [28,29], suggesting an additional mechanism whereby 2HG might affect pre-mRNA splicing.

The evidence indicates that the 1p/19q co-deletion impacts alternative splicing at least in part through its effect on the splicing factor encoded by the *FUBP1* (far upstream element-binding protein 1) gene, located on chromosome 1. Mutations in the remaining allele of *FUBP1* are frequently encountered in oligodendrogliomas carrying the 1p/19q co-deletion [30]. A recent study of the somatic mutational landscape of splicing factor genes across 33 cancer types revealed a significant association of loss-of-function (LoF) mutations affecting the remaining allele of *FUBP1* with alternative splicing and gene downregulation in oligodendrogliomas [31].

Here, we have carried out a bioinformatics analysis to examine the impact of the *IDH* mutation, as well as that of the 1p/19q co-deletion, the G-CIMP status and *FUBP1* status, on alternative splicing and protein isoform expression in a cohort of 662 diffuse gliomas from The Cancer Genome Atlas (TCGA). 

## 2. Results

### 2.1. Splicing Alterations in Gliomas

**Figure 1:**
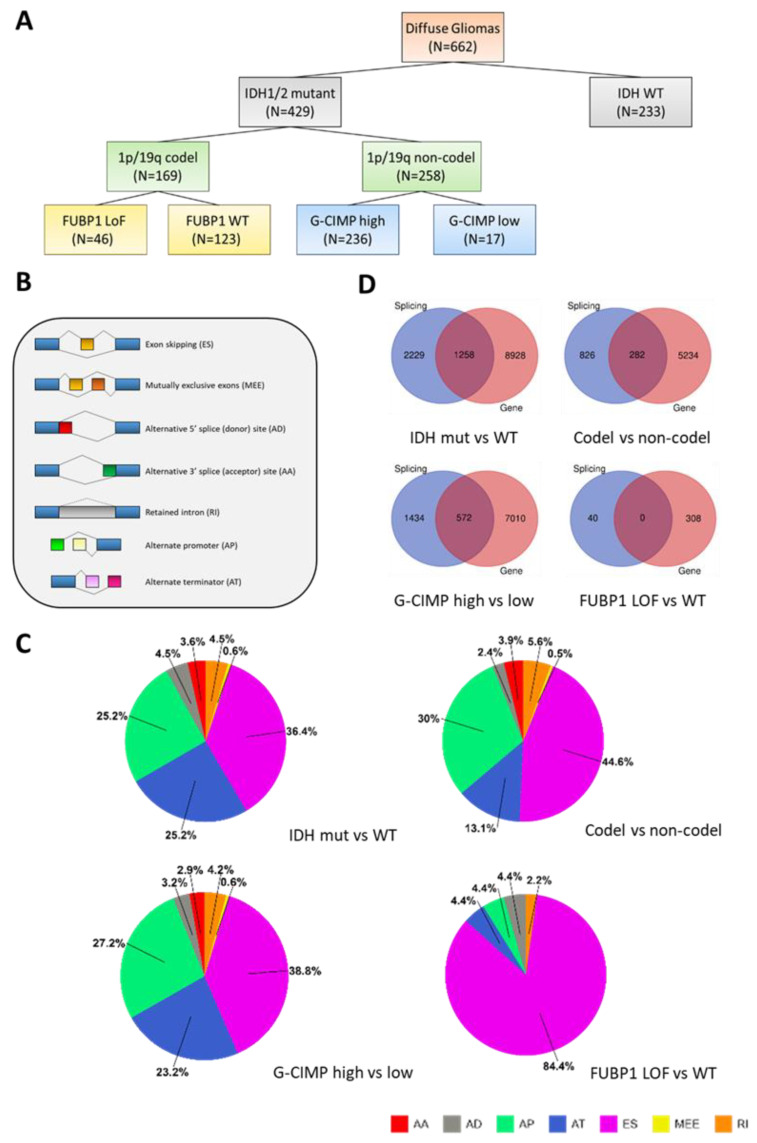
(**A**). Description of the cohort used for the analysis of alternative splicing in diffuse gliomas. (**B**,**C**) Splicing alterations observed in the various comparisons. (**B**) Differential splicing events examined in this study. (**C**) Incidence of the differential splicing events in the indicated comparisons. (**D**) Venn diagrams illustrating the numbers of genes affected at the level of splicing or gene expression for the indicated comparisons.

To explore the impact of mutant IDH, as well as that of the 1p/19q co-deletion and the G-CIMP status, on alternative splicing in gliomas, we undertook an investigation of the transcriptome RNA sequencing (RNAseq) data of a cohort of 662 diffuse gliomas (see Materials and Methods, Section 4.1) contained in The Cancer Genome Atlas (TCGA, https://cancergenome.nih.gov/, accessed on 6 September 2022)) [32]. The samples of this cohort were segregated into the following groups: IDH-wild-type (WT) gliomas, IDH-mutant + 1p/19q non-codel gliomas, and IDH-mutant + 1p/19q codel gliomas. The latter group was further divided into FUBP1 LoF and FUBP1-WT subgroups [31], whereas the 1p/19q non-codel group was divided into G-CIMP-high and G-CIMP-low subgroups (Figure 1A). Notably, we did not identify *FUBP1* mutations in the IDH-WT group (see Supplementary Figure S5A). 

We then used the percent spliced in index (PSI; see Materials and Methods, Section 4.1) to examine the efficiency of exon splicing into the transcript population of a given gene in the following comparisons: IDH-mutant vs. IDH-WT, 1p/19q codel vs. non-codel, G-CIMP-high vs. G-CIMP-low and FUBP1 LoF vs. FUBP1-WT (Figure 1B,C). Strikingly, more than 80% of the differential splicing events associated with the comparison of FUBP1 LoF vs. FUBP1-WT were exon skipping, which is in agreement with the documented role of FUBP1 in regulating exon splicing and modulating exon inclusion [31,33,34,35], as well as its role in shaping the splicing landscape in low-grade gliomas [31]. However, no functional network could be identified from the list of genes affected by exon skipping for this comparison. In the other comparisons, the most frequently encountered differential splicing alterations again included exon skipping events, as well as alternate promoter (alternative first exon) and alternate terminator (alternative last exon) events, which together composed 80% of the observed events (Figure 1C).

Next, we compared the lists of differentially spliced genes obtained above with the lists of differentially expressed genes (DEGs; see Materials and Methods, Section 4.2) which were obtained for the same comparisons (Figure 1D). Supplementary Table S1 provides detailed lists of the alternative splicing (AS) events in all of the genes we found affected either by alternative splicing and differential gene expression (list 1), or by alternative splicing only (list 2), within the four comparisons. For the remaining part of this work, we decided to focus on those genes that were affected by alternative splicing but not by differential gene expression (Supplementary Table S1, list 2).

We first examined the chromosomal distribution of the genes with AS events for the four comparisons, using gene feature annotations from the Molecular Signatures Database (MSigDB) [36], as well as feature annotations from the NCBI gene_info file (ftp://ftp.ncbi.nih.gov/gene/DATA/GENE_INFO/Mammalia/Homo_sapiens.gene_info.gz, accessed on 1 April 2023) (see Materials and Methods, Section 4.3). In the IDH-mutant vs. -WT comparison, chromosome 10 possessed significantly fewer alternative splicing (AS) events than other chromosomes, while chromosome 19, and to a lesser extent, chromosomes 3, 11, 12 and 17, were enriched for AS (Supplementary Figure S1A). In the 1p/19q non-codel vs. codel comparison, chromosomes with enriched AS events included chromosomes 3, 7, 12, 17 and 19. In contrast, chromosome 1 displayed significantly fewer AS events (Supplementary Figure S1B). These observations suggest that, unlike for chromosome 19 which appears relatively unaffected by the 1p/19q co-deletion, an important portion of the genes affected by alternative splicing on chromosome 1 is present on the p arm of this chromosome. In the G-CIMP-high vs. G-CIMP comparison, where all samples have retained 1p and 19q, both chromosomes 1 and 19, as well as chromosomes 12, 16 and 17, were significantly enriched for AS, while several chromosomes displayed slightly fewer AS events than expected (Supplementary Figure S1C). Finally, in the FUBP1 LoF vs. FUBP1-WT comparison of 1p/19q codel samples, we did not observe significant differences in the genomic location of genes affected by AS compared to other genes, which was likely due to the low number of events (40 alternatively spliced genes) (Supplementary Figure S1D). 

In summary, our data suggest that chromosome arm 1p, but not 19q, is significantly affected at the AS level by the 1p/19q co-deletion. Although *FUBP1* is located on chromosome 1p, and while we cannot exclude a role for this factor in the splicing of a subset of the genes located on this chromosome arm, our data also suggest that the loss of AS events on this chromosome arm in the 1p/19q codel subgroup is not the result of *FUBP1* loss of function. Finally, our observations of chromosome 19 suggest that the loss of one chromosome 19q arm in the 1p/19q codel subgroup triggers “compensatory” AS events on the intact arm, presumably participating in the development of these tumors. 

We next examined the distribution of AS events as a function of the number of exons in the affected genes (see Materials and Methods, Section 4.3). The data reported in Supplementary Figure S2 indicate that, for all comparisons, fewer AS events than expected based on ShinyGo [37] were observed for genes composed of less than four exons, whereas they tended to be enriched for genes above four exons, with maximum enrichments observed for genes with five and six exons. Finally, we examined the distribution of AS events as a function of the number of transcripts associated with the differentially spliced genes. Overall, our analysis revealed significantly higher numbers of AS genes than predicted for genes expressing more than six transcripts (Supplementary Figure S3). 

Focusing on AP and AT, we next attempted to trace the AS events identified across the four comparisons (IDH-mutant vs. IDH-WT, 1p/19q codel vs. non-codel, G-CIMP-high vs. G-CIMP-low and FUBP1 LoF vs. FUBP1-WT) to specific molecular alterations (*IDH* mutations, 1p/19q co-deletion and G-CIMP-high). To this end, we carried out a Venn diagram analysis of the data from Supplementary Table S1 (list 2) to identify shared as well as unique AP and AT events within each comparison (Supplementary Figure S4A). Such analysis allowed us to identify AS events associated with the *IDH* mutational status and which (i) remained unchanged in the subsequent subgroup ramifications, or (ii) were further deregulated in one or several subgroups, as well as (iii) AS events unaffected by the *IDH* mutations, but associated with one or several subgroups. Such AS events and their affected genes are represented in Supplementary Figure S4B and listed in Supplementary Table S2. Only three genes were affected by AS events associated with the FUBP1 LoF vs. FUBP1-WT comparison, likely because of the small number of FUBP1 LoF specimens in our cohort. In total, we identified 207 and 327 genes linked to IDH-mutant-independent AS events (AP or AT) in the 1p/19q codel vs. non-codel and G-CIMP-high vs. G-CIMP-low comparisons, respectively. In contrast, 748 genes were linked to AS events associated solely with the *IDH* mutational status, which appear to remain fixed in the subgroup ramifications.

### 2.2. Biological Processes and Molecular Functions Affected by Alternative Splicing in Gliomas

**Figure 2:**
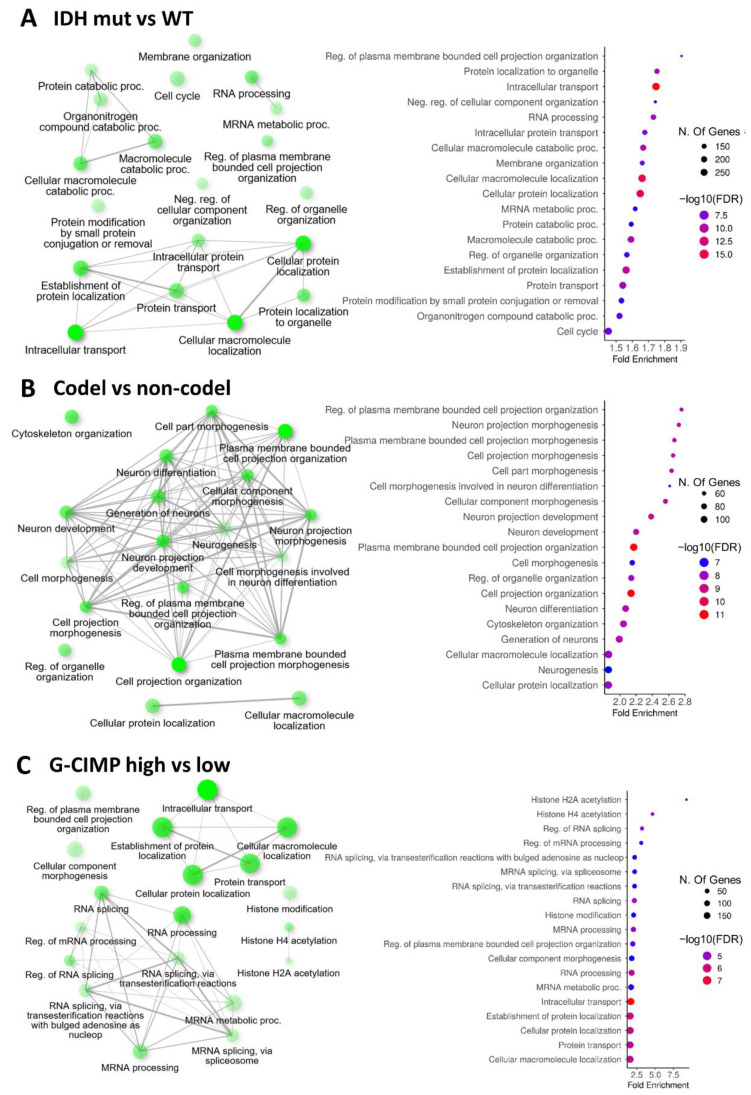
Gene Ontology analyses of the alternatively spliced genes in each clinical group. (**A**–**C**) Representation of the GO biological processes associated with the differentially spliced genes identified in the indicated comparisons. Left panel: Networks of functionally related significant GO biological processes. Individual nodes represent an enriched GO term. Edges represent related terms, and thickness reflects percent of overlapping genes. The size of the node corresponds to number of genes. Right panel: Dot plot showing the fold enrichment of the top significant biological processes, size of the dots (number of genes) and color of dots (−log10 FDR).

To explore the effect of splicing changes on the biology of the various glioma subgroups, we next performed a gene ontology (GO) analysis using ShinyGo [37] on the genes with alternative splicing events identified in the four comparisons (see Materials and Methods, Section 4.3). When biological processes were considered, we found that terms related to intracellular transport, protein/macromolecule localization and RNA processing were enriched in the IDH-mutant vs. IDH-WT comparison (Figure 2A). Remarkably, the 1p/19q codel vs. non-codel comparison revealed a strong enrichment of terms related to neuron morphogenesis, differentiation and development (Figure 2B). Although we found that intracellular transport and protein/macromolecule localization were enriched in the G-CIMP-high vs. G-CIMP-low comparison, the G-CIMP status also appeared to affect unique biological processes at the splicing level. These included histone acetylation, as well as RNA splicing itself (Figure 2C), the latter being illustrated by the presence of several splicing factors among the differentially spliced genes identified for this comparison (Table 1).

Previous studies have associated the alternative splicing of arginine–serine-rich (SR) splicing factors to nonsense-mediated mRNA decay [38]. However, this form of unproductive splicing cannot be the reason for our observations, as we analyzed solely alternatively spliced genes that are not affected at the level of gene expression. No significant enrichments were observed for the FUBP1 LoF vs. FUBP1-WT comparison.

**Figure 3:**
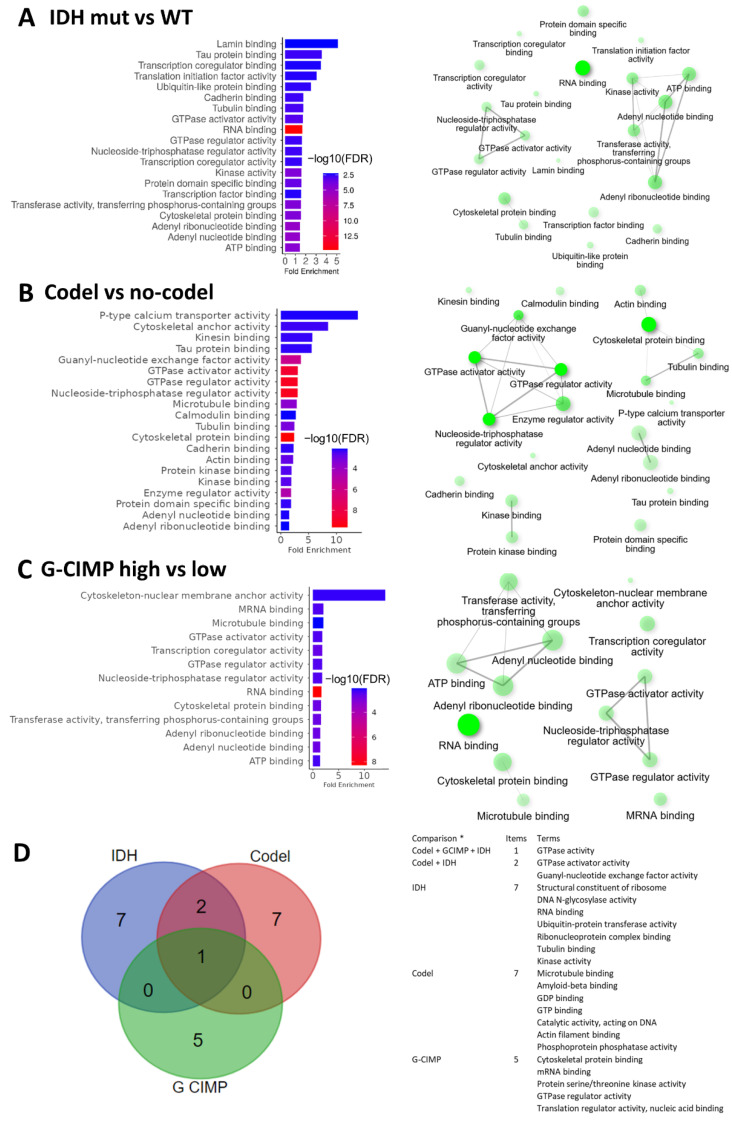
Representation of the GO molecular functions associated with the differentially spliced genes in the indicated comparisons. (**A**–**C**) Left panel: Bar plots showing the fold enrichment of the top significant molecular functions; bar color indicates significance of fold enrichment (-log10 FDR). Right Panel: Networks of functionally related GO molecular functions. Individual nodes represent enriched GO terms. Edges represent related terms, and thickness reflects percent of overlapping genes. The size of the node corresponds to number of genes. (**D**) Left panel: Venn diagram illustrating the number of molecular function terms associated with the differentially spliced genes identified and found in the various comparisons. Right panel: List of the terms. * IDH: IDH-mutant vs. -WT; CODEL: 1p/19q codel vs. non-codel; GCIMP: G-CIMP-high vs. G-CIMP-low.

We next considered the GO molecular functions. We identified that the differentially spliced genes were linked to significant molecular functions in each comparison. We found that mRNA-binding and transcription coregulatory activity were enriched in IDH-mutant vs. IDH-WT (Figure 3A) and in G-CIMP-high vs. G-CIMP-low group comparisons (Figure 3C), but not in the 1p/19q codel vs. non-codel comparison (Figure 3B). This may indicate that the presence of transcript isoforms may further impact specific signal transduction of RNAs. Another striking feature of all comparisons was the presence of terms related to the small GTPase activity and its regulation (Figure 3A–C). Indeed, of all the molecular function terms found enriched in our comparisons, GTPase activity was the only common one (Figure 3D). A list of the GTPase genes identified in our study is presented in Supplementary Table S3.

### 2.3. Modulation of Epigenetic Regulation by Alternate Splicing in Gliomas

**Figure 4:**
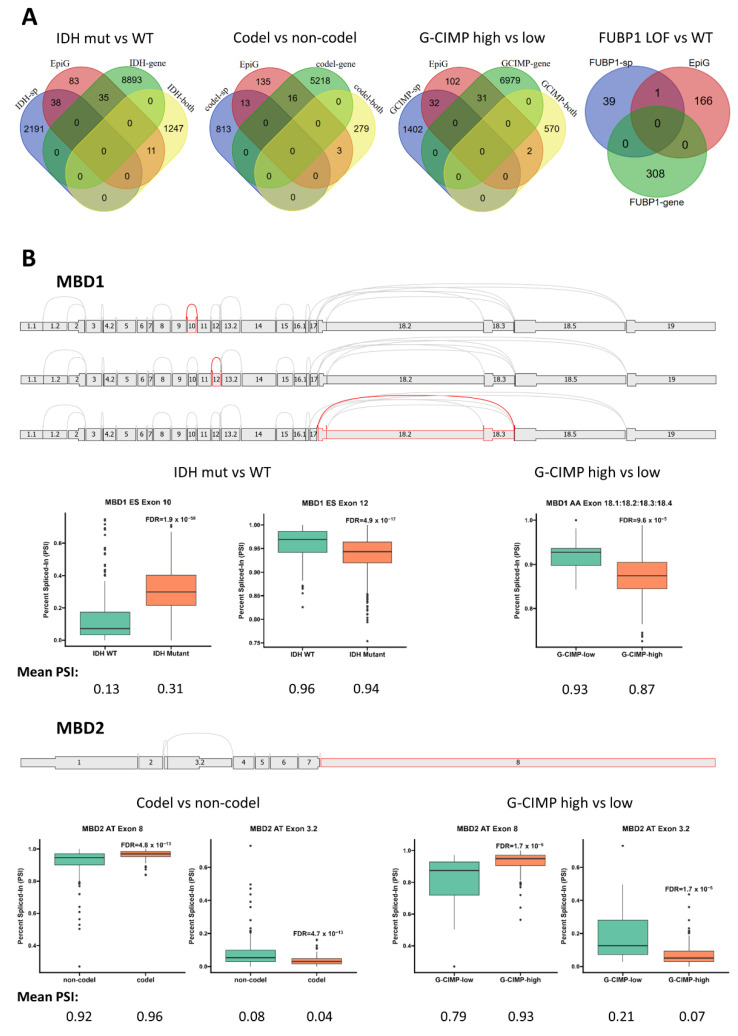
(**A**) Numbers of epigenetic modifiers affected by differential splicing (splice, sp), gene expression (gene) or both in the indicated comparisons. (**B**) Alternative splicing events affecting the *MBD1* and *MBD2* genes in the indicated comparisons. Shown are the distributions of exons in *MBD1* and *MBD2* (grey cassettes), as well as all potential junctions (grey lines). Cassettes with red outlines represent the exons spliced-in in the alternative splicing events detailed in the text, with red lines indicating the junctions in these events. The plots present the PSIs differentially expressed with FDR < 0.05. Splice graphs were generated by SpliceSeq [40].

In a previous comparison of pediatric high-grade glioma (pHGG) samples from the normal brain, Siddaway et al. reported that chromatin modifiers were more affected by splicing than expression changes in pHGG, implying that AS is a crucial modulator of epigenetic regulation in these gliomas [7]. To examine this issue in the diffuse glioma cohort, we compared the lists of differentially spliced and expressed genes identified in our various comparisons with that of the Database of Epigenetic Modifiers (dbEM) [39]. When the IDH-mutant vs. -WT comparison was considered, we found roughly equal numbers of epigenetic modifiers in the genes affected by splicing only (N = 38) or by expression change only (N = 35), with another 11 epigenetic modifiers affected at both levels (Figure 4A). These observations underline the importance of AS in the epigenetic changes brought about by the *IDH* mutations. Notably, differential splicing and gene expression also contributed equally to the deregulation of epigenetic modifiers in the 1p/19q codel vs. non-codel and G-CIMP-high vs. G-CIMP-low comparisons, whereas only one epigenetic modifier was affected in the FUBP1 LoF vs. FUBP1-WT comparison (Figure 4A).

We next examined which epigenetic modifiers were affected by differential splicing in our comparisons (Table 2).

We found that *DNMT3A*, *INO80E*, *MTA1* and *PRMT2* were common to the IDH-mutant vs. -WT, 1p/19q codel vs. non-codel and G-CIMP-high vs. G-CIMP-low comparisons (Table 2). Interestingly, DNA methyltransferase *DNMT3A* and two members of the methyl-CpG-binding domain (MBD) protein family, *MBD1* and MBD2, were among the epigenetic modifiers affected by differential splicing in several comparisons, including the G-CIMP-high vs. G-CIMP-low comparison. Specifically, in the case of *MBD1*, we found that the splicing index of exon 10 was increased in the IDH-mutant glioma (mean PSI: 0.31) compared to IDH-WT (mean PSI: 0.13), indicating the higher expression of an *MBD1* isoform containing this exon in the IDH-mutant glioma. Conversely, a small decrease in the expression of an *MBD1* isoform containing exon 12 was predicted in the IDH-mutant glioma (Figure 4B). *MBD1* splicing variants were also predicted for the G-CIMP-high vs. G-CIMP-low comparison where the percentage of the *MBD1* isoforms containing the exons flanked by exon 17 and 18.5, i.e., 18.1–18.4, was decreased from 93% to 87% (Figure 4B). For *MBD2*, which encodes two isoforms using exon 3.2 and exon 8 as terminators, respectively, we found that usage of exon 8 as the terminator was higher in the 1p/19q codel subgroup (mean PSI: 0.96) than in the non-codel subgroup (mean PSI: 0.92), while the opposite was seen for exon 3.2 (mean PSI: 0.04 vs. 0.08). A similar pattern was observed in the G-CIMP-high vs. G-CIMP comparison, where usage of exon 8 as the terminator was higher in the G-CIMP-high subgroup than in the G-CIMP-low subgroup. 

### 2.4. Glioma Driver Alterations and Alternate Splicing

Having considered the impact of IDH mutations on splicing, we next examined if other mutations that have been implicated as glioma drivers or as clinically relevant markers for subgroup classification were associated with specific alternatively spliced genes. For this study, we examined *EGFR*, *PTEN*, *CIC*, *TP53* and *ATRX*, as well as mutations affecting the *TERT* promoter [30,41,42,43], using the cBioPortal for Cancer Genomics (see Materials and Methods, Section 4.4). The molecular alterations affecting these genes in the 662 specimens of our cohort are illustrated in Supplementary Figure S5A. Notably, *EGFR* alterations (mutations and amplifications, considered together in this study) and *PTEN* alterations (mutations and deep deletions) affected almost exclusively IDH-WT gliomas. On the other hand, mutations affecting *CIC* and *FUBP1* were restricted to the IDH-mutant 1p/19q codel subgroup. ATRX mutations were almost exclusively present in the IDH-mutant 1p/19q non-codel subgroup, and this subgroup also contained the majority of the TP53 mutations. *TERT* promoter mutations were distributed across all subgroups.

We next identified the AS genes associated with *EGFR*, *PTEN*, *CIC*, *TP53, ATRX* and *TERT*. Because alterations affecting these genes were strongly confined to specific subgroups (Supplementary Figure S5A), and to prevent interference from other subgroups, we restricted each analysis to the subgroup(s) hosting the gene alteration under consideration. The identified AS genes are represented in Supplementary Figure S5B and listed in Supplementary Table S4. Very few AS genes were associated with alterations in *EGFR*, *PTEN*, *CIC* and *ATRX.* With the exception of TP53, the GO analysis using DAVID did not reveal any significant enrichment in the biological processes and molecular functions for the identified AS genes associated with the driver genes considered in this work. However, when the mutational status of *TP53* was considered within the IDH-mutant 1p/19q non-codel subgroup, the GO analysis of the AS genes revealed the enrichment of molecular function terms related to small GTPase-binding (GO:0031267; *p*-value 1.4 × 10^−8^, FDR 2.7 × 10^−6^) as well as GTPase-activator activity (GO:0005096; *p*-value 7.28 × 10^−4^, FDR 0.04). 

Finally, the most frequently encountered differential splicing alterations associated with *TP53* mutations in the IDH-mutant 1p/19q non-codel subgroup included exon skipping events (53.1%), whereas alternate promoter (37.1%) and exon skipping (35.3%) predominated in the *TERT*-mutant vs. -WT comparison. Notably, alternate promoter events represented almost two thirds of the AS events in the *EGFR*-mutant vs. -WT comparison, where no alternate terminator event was detected (Supplementary Figure S5C). 

## 3. Discussion

The contribution of alternative splicing to cancer development and biology has received increasing attention in recent years [44,45]. Previous studies have identified alternative splicing as an important oncogenic pathway in high-grade gliomas [7]. In addition, mRNA splicing profiling has been considered as an approach to identify prognostic predictors and potential therapeutic targets for GBM [46]. In this work, we have investigated the impact of *IDH* mutations, the co-deletion of 1p/19q and the G-CIMP phenotype on alternative mRNA splicing and the expression of protein isoforms in diffuse gliomas. 

Our analysis reveals long lists of genes affected at the splicing level by the *IDH* mutations, the co-deletion of 1p/19q and the G-CIMP phenotype. They also suggest that some of the AS events associated with the *IDH* mutations remain fixed in the subsequent ramifications afforded by the 1p/19q codel/non-codel and G-CIMP-high/low phenotypes, while others were specifically associated with these phenotypes. Such analyses should prove a useful resource for readers who want to examine splicing alterations in specific genes and/or clinical subgroups. Notably, several genes and gene families in this list are involved in normal neural/glial function and/or glioma proliferation, adding weight to the notion that pre-mRNA splicing alterations have a significant impact on the biology and behavior of gliomas.

Strikingly, GTPase activity and its regulation are crucial molecular functions affected by alternative splicing in diffuse gliomas. GTPases are small GTP-binding proteins that operate as molecular switches in cytoskeletal dynamics, cell communication, intracellular trafficking and cell migration [47]. GTPases exist in either an active GTP-bound state, during which the GTPase can interact with downstream effectors, or an inactive GDP-bound state. Cycling between these two states is regulated by guanine nucleotide exchange factors, GTPase-activating proteins and guanine nucleotide dissociation inhibitors [48,49]. Small GTPase genes play a crucial role in brain development [50,51]. Small GTPases have been found to be involved in GBM motility, invasion and progression [52,53]. In addition, the evidence indicates that Rho family GTPases modulate DNA repair and resistance to ionizing radiation in WT p53 GBM [54]. GTPases are considered as therapeutic targets in cancer, including gliomas [55,56,57]. The small GTPase family is encoded by dozens of genes displaying complex splicing dynamics [58]. Our data suggest that the repertoire of GTPases and their regulators is subjected to intensive modulation by alternative splicing in key subgroups of diffuse gliomas. How these alternative splicing events contribute to the development, biology, treatment response and prognosis of the various glioma subgroups examined in this study remains to be investigated. In this regard, our data suggest that *TP53* mutations are associated with AS events affecting GTPase-binding and regulation factors in the IDH-mutant 1p/19q non-codel subgroup. Interestingly, altered RNA splicing by the mutant p53, through the modulation of the RNA-binding protein hnRNPK, has been shown to impact GTPase-activating proteins, leading to the activation of oncogenic RAS signaling in pancreatic cancer [59]. Whether the biological processes identified in our analysis reflect a similar role for p53 in splicing regulation and oncogenesis in specific glioma subgroups remains unknown.

Alternative splicing acts as a crucial modulator of epigenetic regulation in pHGG [7]. We found evidence suggesting that this role can be extended to all diffuse gliomas. Remarkably, among the epigenetic modifiers affected by differential splicing in our comparisons were DNA methyltransferases and epigenetic readers of DNA methylation. Although the functional impact of the alterations conferred by the differential splicing of these factors remains to be determined, these observations suggest that the *IDH* mutations elicit an integrated reprogramming that affects not only DNA methylation patterns, but also the expression of variant epigenetic readers of DNA methylation.

Several strategies targeting splicing mechanisms, splicing factors or alternative splicing isoforms are being considered in relation to cancer therapy [44,45]. Our analyses reveal that a great number of genes involved in glioma-relevant biological pathways are affected by alternative splicing in diffuse gliomas. Targeting these genes might provide novel therapeutic opportunities against gliomas.

## 4. Materials and Methods

### 4.1. Differential Splicing Analysis

We downloaded the PSI data pertaining to the diffuse glioma cohort (670 patients with diffuse glioma, including 515 low-grade glioma (LGG) and 155 glioblastoma (GBM), from the TCGA SpliceSeq database (https://bioinformatics.mdanderson.org/TCGASpliceSeq/PSIdownload.jsp, accessed on 6 September 2022) [60] as well as that of molecular subtypes (clinical subgroup, i.e., IDH status, 1p/19q co-deletion and Glioma CpG island methylator phenotype) of the TCGA samples using the *TCGAquery_subtype* function of the TCGAbiolinks R package (10.1371/journal.pcbi.1006701) and the FUBP1 mutation data from the TCGA MC3 working group [61] using the *getMC3MAF* function of the TCGAbiolinks R package. Then, 8 samples with IDH information missing were removed and the data from the remaining 662 samples were used for further analysis. We performed differential expression analysis according to the methods described by Seiler et al. [31]. Briefly, we filtered out the splicing sites which had a 0 PSI in more than half of the samples from both groups in the comparison. We converted each PSI to log odds (log(PSI/(1-PSI))), and then fit a linear model for each splicing site and computed the moderate t-statistic to define differential expression using the limma R package [62]. We considered the splicing sites with adjusted *p*-values (FDR: false discovery rate) < 0.05 and log2 fold changes (FC) > 0.5 or <−0.5 as significantly regulated for all types of splicing events. 

### 4.2. Differential Gene Expression Analysis

We downloaded the RNA-seq raw counts of the GBM (N = 173) and LGG (N = 529) samples from the TCGA database (https://portal.gdc.cancer.gov/, accessed on 4 February 2018). We kept the data from 656 primary tumor samples, which also had PSI data, for further analysis. We filtered out the genes detected with less than 10 reads in more than half of the samples. We then used the functions *DESeq* and *results* from the DESeq2 R package [63] to perform size factor estimation, dispersion estimation and negative binomial GLM fitting sequentially for differentially expressed gene identification. We considered the genes with an FDR < 0.05 and a log2 FC change > 0.5 or <−0.5 to be differentially expressed.

### 4.3. Over- and Under-Representation Analyses and Pathway Enrichment

In general, the over-representation analysis was conducted using ShinyGO 0.77 (http://bioinformatics.sdstate.edu/go, accessed on 30 March 2023/) [37], which allowed for direct visual representation, or DAVID (Database for Annotation, Visualization and Integrated Discovery) [64,65]. For diagrams we used Pathview [66] with KEGG database pathway annotation [67]. A cut-off of an FDR < 0.01 was applied for significance. For Supplementary Figure S1, the over- or under-representation of individual chromosomes was analyzed using the hypergeometric test. Gene locations were obtained with feature annotations from the NCBI database (ftp://ftp.ncbi.nih.gov/gene/DATA/GENE_INFO/Mammalia/Homo_sapiens.gene_info.gz, accessed on 23 May 2023). Analysis was performed in R software (R.4.2.3) using the *phyper* function, where the lower.tail parameter was set to TRUE for under-representation and FALSE for lower over-representation. *p*-values were adjusted for multiple testing errors using the Benjamini–Hochberg procedure [68]. All genes present in the database were considered as the background data. 

### 4.4. Oncoprint Generation

The oncoprint of Supplementary Figure S5A was generated by searching glioma studies in the cBioPortal for Cancer Genomics (https://www.cbioportal.org/, accessed on 1 May 2023) [64,65].

## Figures and Tables

**Table 1:** List of splicing factors affected by AS in the G-CIMP-high vs. G-CIMP-low comparison.

CELF1 (CUGBP Elav-like family member 1)
CELF2 (CUGBP Elav-like family member 2)
CELF3 (CUGBP Elav-like family member 3)
CELF6 (CUGBP Elav-like family member 6)
CIRBP (cold inducible RNA binding protein)
DAZAP1 (DAZ associated protein 1)
ISY1 (ISY1 splicing factor homolog)
LUC7L (LUC7 like)
LUC7L3 (LUC7 like 3 pre-mRNA splicing factor)
PUF60 (poly(U) binding splicing factor 60)
QKI (QKI, KH domain containing RNA binding)
SF1 (splicing factor 1)
SMN1 (survival of motor neuron 1, telomeric)
SMN2 (survival of motor neuron 2, centromeric)

**Table 2:** Epigenetic modifiers affected by AS in the indicated comparisons. * IDH: IDH-mutant vs. -WT; CODEL: 1p/19q codel vs. non-codel; GCIMP: G-CIMP-high vs. G-CIMP-low; FUBP: FUBP1 LoF vs. FUBP1-WT.

Comparison *	Total	Elements	Human Symbol
CODEL + GCIMP + IDH	4	INO80E	INO80E (INO80 complex subunit E)
		MTA1	MTA1 (metastasis associated 1)
		DNMT3A	DNMT3A (DNA methyltransferase 3 alpha)
		PRMT2	PRMT2 (protein arginine methyltransferase 2)
FUBP1 + GCIMP + IDH	1	ELP3	ELP3 (elongator acetyltransferase complex subunit 3)
GCIMP + IDH	15	SIRT2	SIRT2 (sirtuin 2)
		CARM1	CARM1 (coactivator associated arginine methyltransferase 1)
		SMARCA4	SMARCA4 (SWI/SNF related, matrix associated, actin dependent regulator of chromatin, subfamily a, member 4)
		EHMT1	EHMT1 (euchromatic histone lysine methyltransferase 1)
		SUV420H2	KMT5C (lysine methyltransferase 5C)
		KDM2B	KDM2B (lysine demethylase 2B)
		JMJD6	JMJD6 (jumonji domain containing 6, arginine demethylase and lysine hydroxylase)
		MCRS1	MCRS1 (microspherule protein 1)
		SETD8	KMT5A (lysine methyltransferase 5A)
		RUVBL2	RUVBL2 (RuvB like AAA ATPase 2)
		KDM5C	KDM5C (lysine demethylase 5C)
		HAT1	HAT1 (histone acetyltransferase 1)
		MBD1	MBD1 (methyl-CpG binding domain protein 1)
		SETD4	SETD4 (SET domain containing 4)
		SIRT7	SIRT7 (sirtuin 7)
CODEL + GCIMP	2	MBD2	MBD2 (methyl-CpG binding domain protein 2)
		SETD5	SETD5 (SET domain containing 5)
IDH only	18	RBBP4	RBBP4 (RB binding protein 4, chromatin remodeling factor)
		WHSC1	NSD2 (nuclear receptor binding SET domain protein 2)
		SETD6	SETD6 (SET domain containing 6, protein lysine methyltransferase)
		NSD1	NSD1 (nuclear receptor binding SET domain protein 1)
		HDAC9	HDAC9 (histone deacetylase 9)
		HDAC8	HDAC8 (histone deacetylase 8)
		ING3	ING3 (inhibitor of growth family member 3)
		INO80C	INO80C (INO80 complex subunit C)
		SIRT5	SIRT5 (sirtuin 5)
		PHF8	PHF8 (PHD finger protein 8)
		SMARCD2	SMARCD2 (SWI/SNF related, matrix associated, actin dependent regulator of chromatin, subfamily d, member 2)
		NAT10	NAT10 (N-acetyltransferase 10)
		SUV39H2	SUV39H2 (SUV39H2 histone lysine methyltransferase)
		ING4	ING4 (inhibitor of growth family member 4)
		CDYL	CDYL (chromodomain Y like)
		KDM6A	KDM6A (lysine demethylase 6A)
		SETMAR	SETMAR (SET domain and mariner transposase fusion gene)
		SMYD3	SMYD3 (SET and MYND domain containing 3)
CODEL only	7	KAT6B	KAT6B (lysine acetyltransferase 6B)
		SMARCC2	SMARCC2 (SWI/SNF related, matrix associated, actin dependent regulator of chromatin, subfamily c member 2)
		SETDB2	SETDB2 (SET domain bifurcated histone lysine methyltransferase 2)
		CHD3	CHD3 (chromodomain helicase DNA binding protein 3)
		KMT2C	KMT2C (lysine methyltransferase 2C)
		DNMT1	DNMT1 (DNA methyltransferase 1)
		SUV420H1	KMT5B (lysine methyltransferase 5B)
GCIMP only	10	PRMT1	PRMT1 (protein arginine methyltransferase 1)
		SIRT1	SIRT1 (sirtuin 1)
		KMT2B	KMT2B (lysine methyltransferase 2B)
		MTA3	MTA3 (metastasis associated 1 family member 3)
		PRMT7	PRMT7 (protein arginine methyltransferase 7)
		JARID2	JARID2 (jumonji and AT-rich interaction domain containing 2)
		PRMT3	PRMT3 (protein arginine methyltransferase 3)
		MTA2	MTA2 (metastasis associated 1 family member 2)
		KAT5	KAT5 (lysine acetyltransferase 5)
		HDAC7	HDAC7 (histone deacetylase 7)

## Data Availability

The data used in this work is publicly available at TCGA (https://cancergenome.nih.gov/, accessed on 4 February 2018). The PSI values were downloaded from TCGA SpliceSeq (https://bioinformatics.mdanderson.org/TCGASpliceSeq/PSIdownload.jsp, accessed on 6 September 2022).

## Supplementary Materials

The following supporting information can be downloaded at: https://www.mdpi.com/article/10.3390/ijms24129825/s1.

## References

[1] Reifenberger G., Wirsching H.G., Knobbe-Thomsen C.B., Weller M. (2016). Advances in the molecular genetics of gliomas—Implications for classification and therapy. Nat. Rev. Clin. Oncol..

[2] Noushmehr H., Weisenberger D.J., Diefes K., Phillips H.S., Pujara K., Berman B.P., Pan F., Pelloski C.E., Sulman E.P., Bhat K.P. (2010). Identification of a CpG island methylator phenotype that defines a distinct subgroup of glioma. Cancer Cell.

[3] Kalidindi N., Or R., Babak S., Mason W. (2020). Molecular Classification of Diffuse Gliomas. Can. J. Neurol. Sci..

[4] Malta T.M., de Souza C.F., Sabedot T.S., Silva T.C., Mosella M.S., Kalkanis S.N., Snyder J., Castro A.V.B., Noushmehr H. (2018). Glioma CpG island methylator phenotype (G-CIMP): Biological and clinical implications. Neuro. Oncol..

[5] Labussiere M., Idbaih A., Wang X.W., Marie Y., Boisselier B., Falet C., Paris S., Laffaire J., Carpentier C., Criniere E. (2010). All the 1p19q codeleted gliomas are mutated on IDH1 or IDH2. Neurology.

[6] Turcan S., Rohle D., Goenka A., Walsh L.A., Fang F., Yilmaz E., Campos C., Fabius A.W., Lu C., Ward P.S. (2012). IDH1 mutation is sufficient to establish the glioma hypermethylator phenotype. Nature.

[7] Siddaway R., Milos S., Vadivel A.K.A., Dobson T.H.W., Swaminathan J., Ryall S., Pajovic S., Patel P.G., Nazarian J., Becher O. (2022). Splicing is an alternate oncogenic pathway activation mechanism in glioma. Nat. Commun..

[8] Yan H., Parsons D.W., Jin G., McLendon R., Rasheed B.A., Yuan W., Kos I., Batinic-Haberle I., Jones S., Riggins G.J. (2009). IDH1 and IDH2 mutations in gliomas. N. Engl. J. Med..

[9] Zhang C., Moore L.M., Li X., Yung W.K., Zhang W. (2013). IDH1/2 mutations target a key hallmark of cancer by deregulating cellular metabolism in glioma. Neuro. Oncol..

[10] Yang H., Ye D., Guan K.L., Xiong Y. (2012). IDH1 and IDH2 mutations in tumorigenesis: Mechanistic insights and clinical perspectives. Clin. Cancer Res..

[11] Losman J.A., Kaelin W.G. (2013). What a difference a hydroxyl makes: Mutant IDH, (R)-2-hydroxyglutarate, and cancer. Genes Dev..

[12] Izquierdo-Garcia J.L., Viswanath P., Eriksson P., Chaumeil M.M., Pieper R.O., Phillips J.J., Ronen S.M. (2015). Metabolic reprogramming in mutant IDH1 glioma cells. PLoS ONE.

[13] Fack F., Tardito S., Hochart G., Oudin A., Zheng L., Fritah S., Golebiewska A., Nazarov P.V., Bernard A., Hau A.C. (2017). Altered metabolic landscape in IDH-mutant gliomas affects phospholipid, energy, and oxidative stress pathways. EMBO Mol. Med..

[14] Han S., Liu Y., Cai S.J., Qian M., Ding J., Larion M., Gilbert M.R., Yang C. (2020). IDH mutation in glioma: Molecular mechanisms and potential therapeutic targets. Br. J. Cancer.

[15] Liu A., Hou C., Chen H., Zong X., Zong P. (2016). Genetics and Epigenetics of Glioblastoma: Applications and Overall Incidence of IDH1 Mutation. Front. Oncol..

[16] Kohli R.M., Zhang Y. (2013). TET enzymes, TDG and the dynamics of DNA demethylation. Nature.

[17] Unruh D., Zewde M., Buss A., Drumm M.R., Tran A.N., Scholtens D.M., Horbinski C. (2019). Methylation and transcription patterns are distinct in IDH mutant gliomas compared to other IDH mutant cancers. Sci. Rep..

[18] Nomura M., Saito K., Aihara K., Nagae G., Yamamoto S., Tatsuno K., Ueda H., Fukuda S., Umeda T., Tanaka S. (2019). Publisher Correction: DNA demethylation is associated with malignant progression of lower-grade gliomas. Sci. Rep..

[19] Zheng G., Dahl J.A., Niu Y., Fedorcsak P., Huang C.M., Li C.J., Vagbo C.B., Shi Y., Wang W.L., Song S.H. (2013). ALKBH5 is a mammalian RNA demethylase that impacts RNA metabolism and mouse fertility. Mol. Cell.

[20] Jia G., Fu Y., Zhao X., Dai Q., Zheng G., Yang Y., Yi C., Lindahl T., Pan T., Yang Y.G. (2011). N6-methyladenosine in nuclear RNA is a major substrate of the obesity-associated FTO. Nat. Chem. Biol..

[21] Cao G., Li H.B., Yin Z., Flavell R.A. (2016). Recent advances in dynamic m6A RNA modification. Open Biol..

[22] Lee M., Kim B., Kim V.N. (2014). Emerging roles of RNA modification: M(6)A and U-tail. Cell.

[23] Niu Y., Zhao X., Wu Y.S., Li M.M., Wang X.J., Yang Y.G. (2013). N6-methyl-adenosine (m6A) in RNA: An old modification with a novel epigenetic function. Genom. Proteom. Bioinform..

[24] Liu N., Zhou K.I., Parisien M., Dai Q., Diatchenko L., Pan T. (2017). N6-methyladenosine alters RNA structure to regulate binding of a low-complexity protein. Nucleic Acids Res..

[25] Liu N., Dai Q., Zheng G., He C., Parisien M., Pan T. (2015). N(6)-methyladenosine-dependent RNA structural switches regulate RNA-protein interactions. Nature.

[26] Xiao W., Adhikari S., Dahal U., Chen Y.S., Hao Y.J., Sun B.F., Sun H.Y., Li A., Ping X.L., Lai W.Y. (2016). Nuclear m(6)A Reader YTHDC1 Regulates mRNA Splicing. Mol. Cell.

[27] Zhao X., Yang Y., Sun B.F., Shi Y., Yang X., Xiao W., Hao Y.J., Ping X.L., Chen Y.S., Wang W.J. (2014). FTO-dependent demethylation of N6-methyladenosine regulates mRNA splicing and is required for adipogenesis. Cell Res..

[28] Webby C.J., Wolf A., Gromak N., Dreger M., Kramer H., Kessler B., Nielsen M.L., Schmitz C., Butler D.S., Yates J.R. (2009). Jmjd6 catalyses lysyl-hydroxylation of U2AF65, a protein associated with RNA splicing. Science.

[29] Heim A., Grimm C., Muller U., Haussler S., Mackeen M.M., Merl J., Hauck S.M., Kessler B.M., Schofield C.J., Wolf A. (2014). Jumonji domain containing protein 6 (Jmjd6) modulates splicing and specifically interacts with arginine-serine-rich (RS) domains of SR- and SR-like proteins. Nucleic Acids Res..

[30] Yip S., Butterfield Y.S., Morozova O., Chittaranjan S., Blough M.D., An J., Birol I., Chesnelong C., Chiu R., Chuah E. (2012). Concurrent CIC mutations, IDH mutations, and 1p/19q loss distinguish oligodendrogliomas from other cancers. J. Pathol..

[31] Seiler M., Peng S., Agrawal A.A., Palacino J., Teng T., Zhu P., Smith P.G., Cancer Genome Atlas Research N., Buonamici S., Yu L. (2018). Somatic Mutational Landscape of Splicing Factor Genes and Their Functional Consequences across 33 Cancer Types. Cell Rep..

[32] Grossman R.L., Heath A.P., Ferretti V., Varmus H.E., Lowy D.R., Kibbe W.A., Staudt L.M. (2016). Toward a Shared Vision for Cancer Genomic Data. N. Engl. J. Med..

[33] Jacob A.G., Singh R.K., Mohammad F., Bebee T.W., Chandler D.S. (2014). The splicing factor FUBP1 is required for the efficient splicing of oncogene MDM2 pre-mRNA. J. Biol. Chem..

[34] Li H., Wang Z., Zhou X., Cheng Y., Xie Z., Manley J.L., Feng Y. (2013). Far upstream element-binding protein 1 and RNA secondary structure both mediate second-step splicing repression. Proc. Natl. Acad. Sci. USA.

[35] Elman J.S., Ni T.K., Mengwasser K.E., Jin D., Wronski A., Elledge S.J., Kuperwasser C. (2019). Identification of FUBP1 as a Long Tail Cancer Driver and Widespread Regulator of Tumor Suppressor and Oncogene Alternative Splicing. Cell Rep..

[36] Subramanian A., Tamayo P., Mootha V.K., Mukherjee S., Ebert B.L., Gillette M.A., Paulovich A., Pomeroy S.L., Golub T.R., Lander E.S. (2005). Gene set enrichment analysis: A knowledge-based approach for interpreting genome-wide expression profiles. Proc. Natl. Acad. Sci. USA.

[37] Ge S.X., Jung D., Yao R. (2020). ShinyGO: A graphical gene-set enrichment tool for animals and plants. Bioinformatics.

[38] Lareau L.F., Brenner S.E. (2015). Regulation of splicing factors by alternative splicing and NMD is conserved between kingdoms yet evolutionarily flexible. Mol. Biol. Evol..

[39] Singh Nanda J., Kumar R., Raghava G.P. (2016). dbEM: A database of epigenetic modifiers curated from cancerous and normal genomes. Sci. Rep..

[40] Ryan M.C., Cleland J., Kim R., Wong W.C., Weinstein J.N. (2012). SpliceSeq: A resource for analysis and visualization of RNA-Seq data on alternative splicing and its functional impacts. Bioinformatics.

[41] Crespo I., Vital A.L., Gonzalez-Tablas M., Patino Mdel C., Otero A., Lopes M.C., de Oliveira C., Domingues P., Orfao A., Tabernero M.D. (2015). Molecular and Genomic Alterations in Glioblastoma Multiforme. Am. J. Pathol..

[42] Cancer Genome Atlas Research N. (2008). Comprehensive genomic characterization defines human glioblastoma genes and core pathways. Nature.

[43] Cancer Genome Atlas Research N., Brat D.J., Verhaak R.G., Aldape K.D., Yung W.K., Salama S.R., Cooper L.A., Rheinbay E., Miller C.R., Vitucci M. (2015). Comprehensive, Integrative Genomic Analysis of Diffuse Lower-Grade Gliomas. N. Engl. J. Med..

[44] Zhang Y., Qian J., Gu C., Yang Y. (2021). Alternative splicing and cancer: A systematic review. Signal. Transduct. Target. Ther..

[45] Ouyang J., Zhang Y., Xiong F., Zhang S., Gong Z., Yan Q., He Y., Wei F., Zhang W., Zhou M. (2021). The role of alternative splicing in human cancer progression. Am. J. Cancer Res..

[46] Zhang B., Wu Q., Cheng S., Li W. (2021). Systematic Profiling of mRNA Splicing Reveals the Prognostic Predictor and Potential Therapeutic Target for Glioblastoma Multiforme. J. Oncol..

[47] Crosas-Molist E., Samain R., Kohlhammer L., Orgaz J.L., George S.L., Maiques O., Barcelo J., Sanz-Moreno V. (2022). Rho GTPase signaling in cancer progression and dissemination. Physiol. Rev..

[48] Jaffe A.B., Hall A. (2005). Rho GTPases: Biochemistry and biology. Annu Rev. Cell Dev. Biol..

[49] Hodge R.G., Ridley A.J. (2016). Regulating Rho GTPases and their regulators. Nat. Rev. Mol. Cell Biol..

[50] Gonzalez-Billault C., Munoz-Llancao P., Henriquez D.R., Wojnacki J., Conde C., Caceres A. (2012). The role of small GTPases in neuronal morphogenesis and polarity. Cytoskeleton.

[51] Zamboni V., Jones R., Umbach A., Ammoni A., Passafaro M., Hirsch E., Merlo G.R. (2018). Rho GTPases in Intellectual Disability: From Genetics to Therapeutic Opportunities. Int. J. Mol. Sci..

[52] Al-Koussa H., Atat O.E., Jaafar L., Tashjian H., El-Sibai M. (2020). The Role of Rho GTPases in Motility and Invasion of Glioblastoma Cells. Anal. Cell Pathol..

[53] Fortin Ensign S.P., Mathews I.T., Symons M.H., Berens M.E., Tran N.L. (2013). Implications of Rho GTPase Signaling in Glioma Cell Invasion and Tumor Progression. Front. Oncol..

[54] Magalhaes Y.T., Boell V.K., Cardella G.D., Forti F.L. (2023). Downregulation of the Rho GTPase pathway abrogates resistance to ionizing radiation in wild-type p53 glioblastoma by suppressing DNA repair mechanisms. Cell Death Dis..

[55] Sheng K.L., Pridham K.J., Sheng Z., Lamouille S., Varghese R.T. (2018). Functional Blockade of Small GTPase RAN Inhibits Glioblastoma Cell Viability. Front. Oncol..

[56] Cemeli T., Guasch-Valles M., Ribes-Santolaria M., Ibars E., Navaridas R., Dolcet X., Pedraza N., Colomina N., Torres-Rosell J., Ferrezuelo F. (2022). Antitumor Effects of Ral-GTPases Downregulation in Glioblastoma. Int. J. Mol. Sci..

[57] Clayton N.S., Ridley A.J. (2020). Targeting Rho GTPase Signaling Networks in Cancer. Front. Cell Dev. Biol..

[58] Das A.S., Sherry E.C., Vaughan R.M., Henderson M.L., Zieba J., Uhl K.L., Koehn O., Bupp C.P., Rajasekaran S., Li X. (2022). The complex, dynamic SpliceOme of the small GTPase transcripts altered by technique, sex, genetics, tissue specificity, and RNA base editing. Front. Cell Dev. Biol..

[59] Escobar-Hoyos L.F., Penson A., Kannan R., Cho H., Pan C.H., Singh R.K., Apken L.H., Hobbs G.A., Luo R., Lecomte N. (2020). Altered RNA Splicing by Mutant p53 Activates Oncogenic RAS Signaling in Pancreatic Cancer. Cancer Cell.

[60] Ryan M., Wong W.C., Brown R., Akbani R., Su X., Broom B., Melott J., Weinstein J. (2016). TCGASpliceSeq a compendium of alternative mRNA splicing in cancer. Nucleic Acids Res..

[61] Ellrott K., Bailey M.H., Saksena G., Covington K.R., Kandoth C., Stewart C., Hess J., Ma S., Chiotti K.E., McLellan M. (2018). Scalable Open Science Approach for Mutation Calling of Tumor Exomes Using Multiple Genomic Pipelines. Cell Syst..

[62] Ritchie M.E., Phipson B., Wu D., Hu Y., Law C.W., Shi W., Smyth G.K. (2015). limma powers differential expression analyses for RNA-sequencing and microarray studies. Nucleic Acids Res..

[63] Love M.I., Huber W., Anders S. (2014). Moderated estimation of fold change and dispersion for RNA-seq data with DESeq2. Genome. Biol..

[64] Cerami E., Gao J., Dogrusoz U., Gross B.E., Sumer S.O., Aksoy B.A., Jacobsen A., Byrne C.J., Heuer M.L., Larsson E. (2012). The cBio cancer genomics portal: An open platform for exploring multidimensional cancer genomics data. Cancer Discov..

[65] Gao J., Aksoy B.A., Dogrusoz U., Dresdner G., Gross B., Sumer S.O., Sun Y., Jacobsen A., Sinha R., Larsson E. (2013). Integrative analysis of complex cancer genomics and clinical profiles using the cBioPortal. Sci. Signal..

[66] Luo W., Brouwer C. (2013). Pathview: An R/Bioconductor package for pathway-based data integration and visualization. Bioinformatics.

[67] Kanehisa M., Furumichi M., Sato Y., Ishiguro-Watanabe M., Tanabe M. (2021). KEGG: Integrating viruses and cellular organisms. Nucleic Acids Res..

[68] Benjamini Y., Hochberg Y. (1995). Controlling the False Discovery Rate: A Practical and Powerful Approach to Multiple Testing. J. R. Stat. Soc. Ser. B (Methodol.).

