# Supplementary material for: Impact of IDH Mutations, the 1p/19q Co-Deletion and the G-CIMP Status on Alternative Splicing in Diffuse Gliomas
Source: Int J Mol Sci. 2023 Jun 6;24(12):9825. doi: 10.3390/ijms24129825 (PMC10297931; doi:10.3390/ijms24129825)
Supplement: Supplementary file 1 [file ijms-24-09825-s001.zip › Zhang et al - Supp Fig 1-3 - IJMB- 1June23.pptx]

## Slide 1
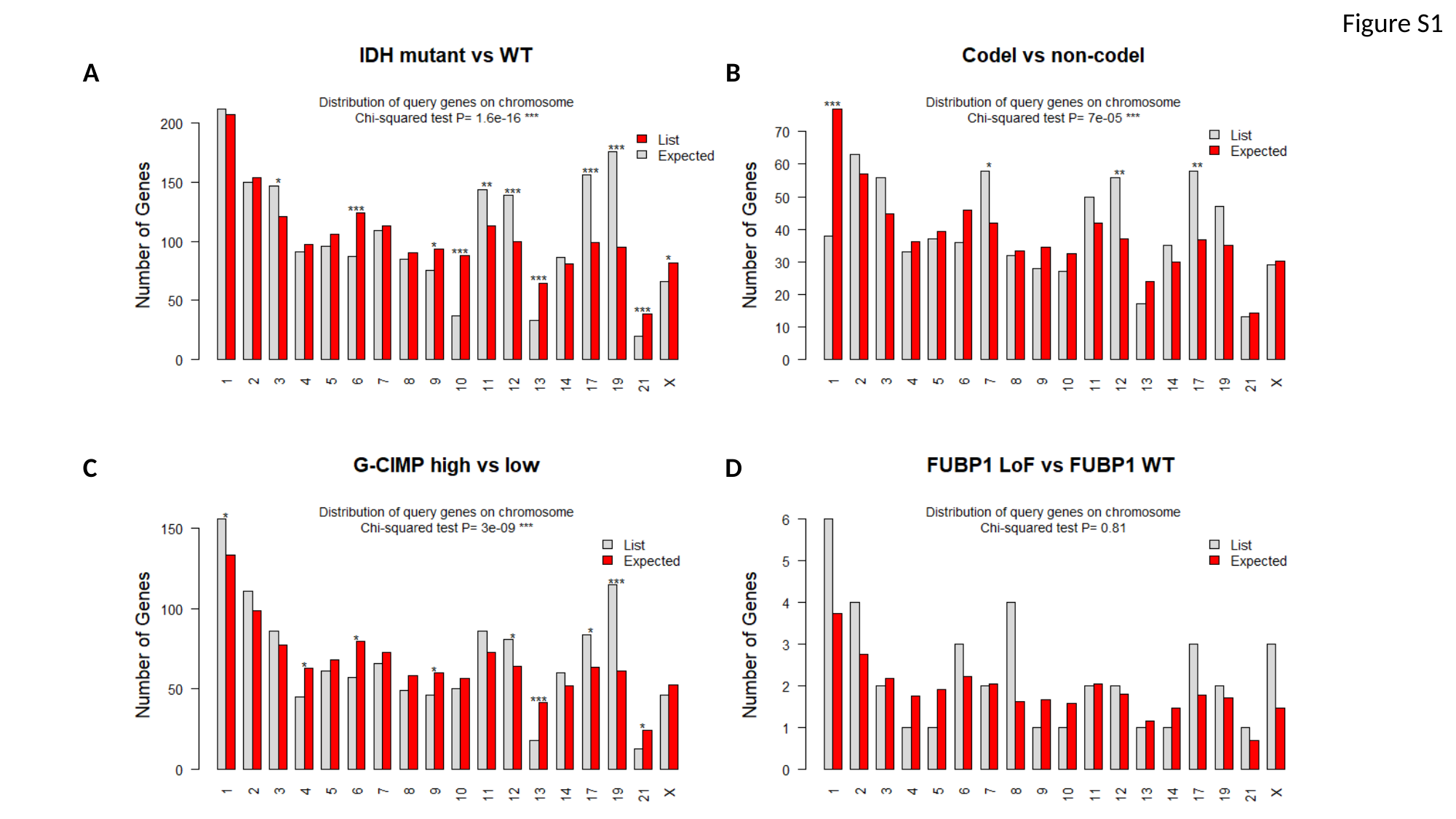

Figure S1
A
B
C
D

## Slide 2
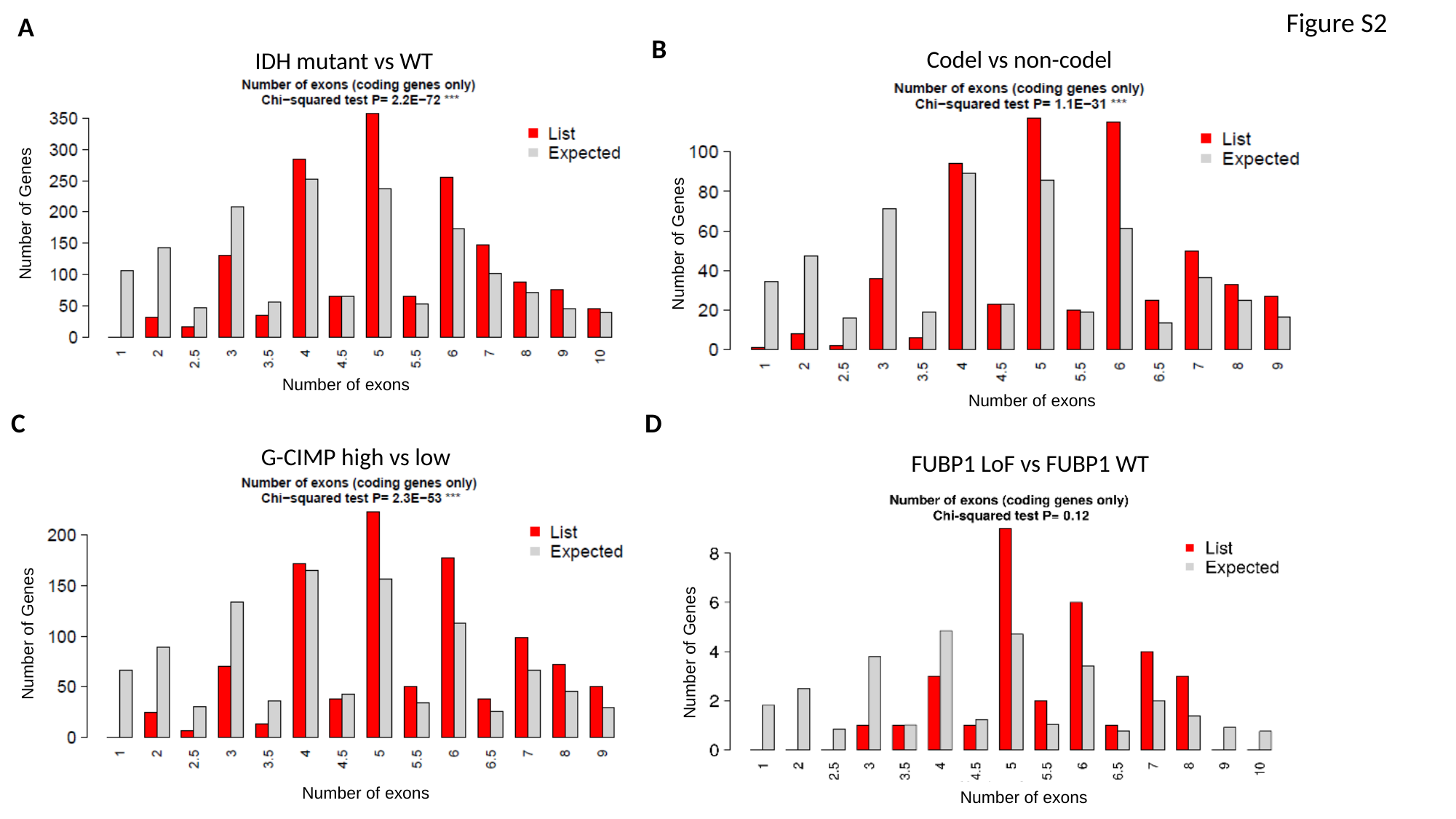

Figure S2
A
B
Codel vs non-codel
IDH mutant vs WT
Number of Genes
Number of Genes
Number of exons
Number of exons
C
D
G-CIMP high vs low
FUBP1 LoF vs FUBP1 WT
Number of Genes
Number of Genes
Number of exons
Number of exons

## Slide 3
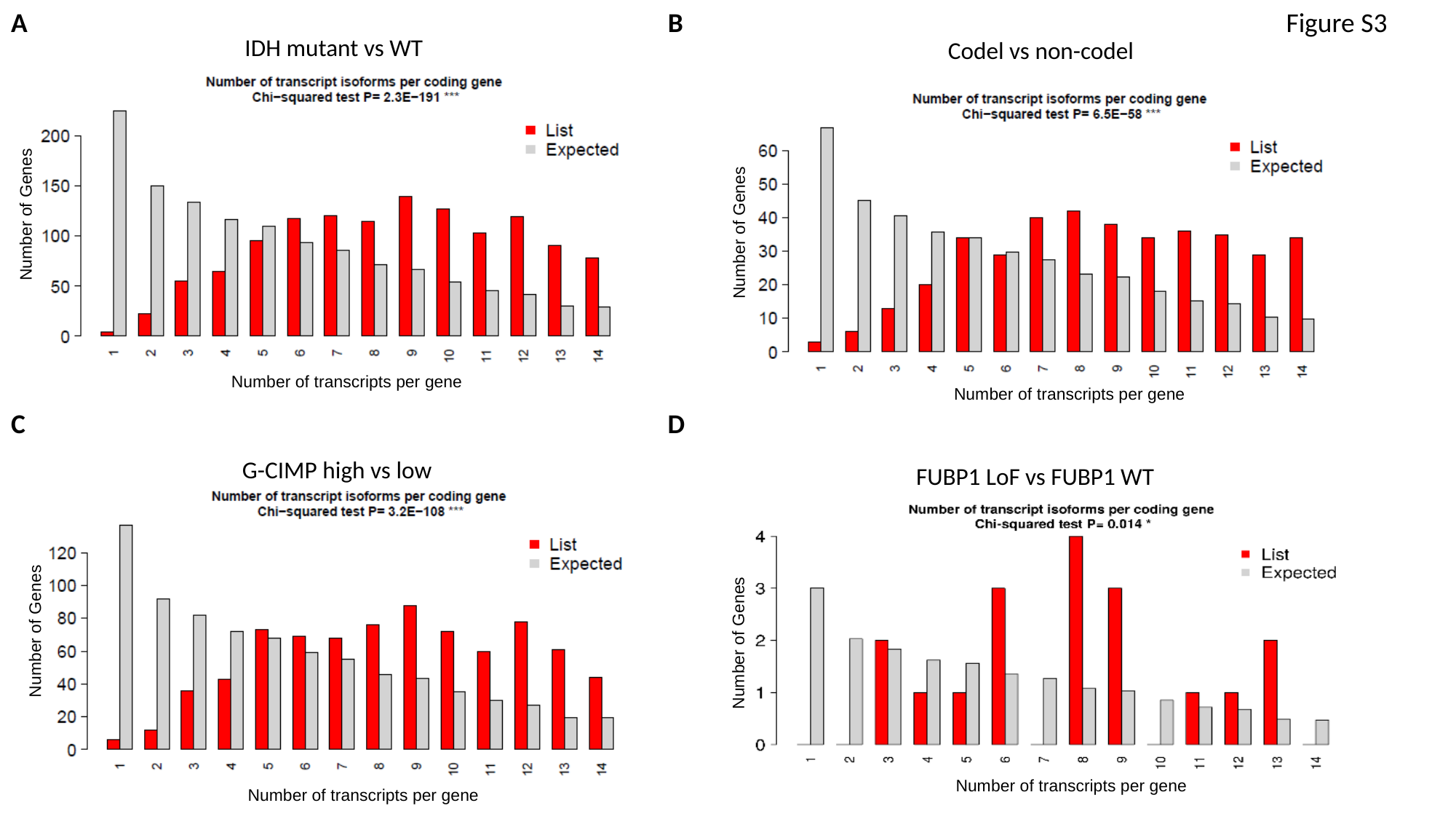

A
B
Figure S3
IDH mutant vs WT
Codel vs non-codel
Number of Genes
Number of Genes
Number of transcripts per gene
Number of transcripts per gene
C
D
G-CIMP high vs low
FUBP1 LoF vs FUBP1 WT
Number of Genes
Number of Genes
Number of transcripts per gene
Number of transcripts per gene

## Slide 4
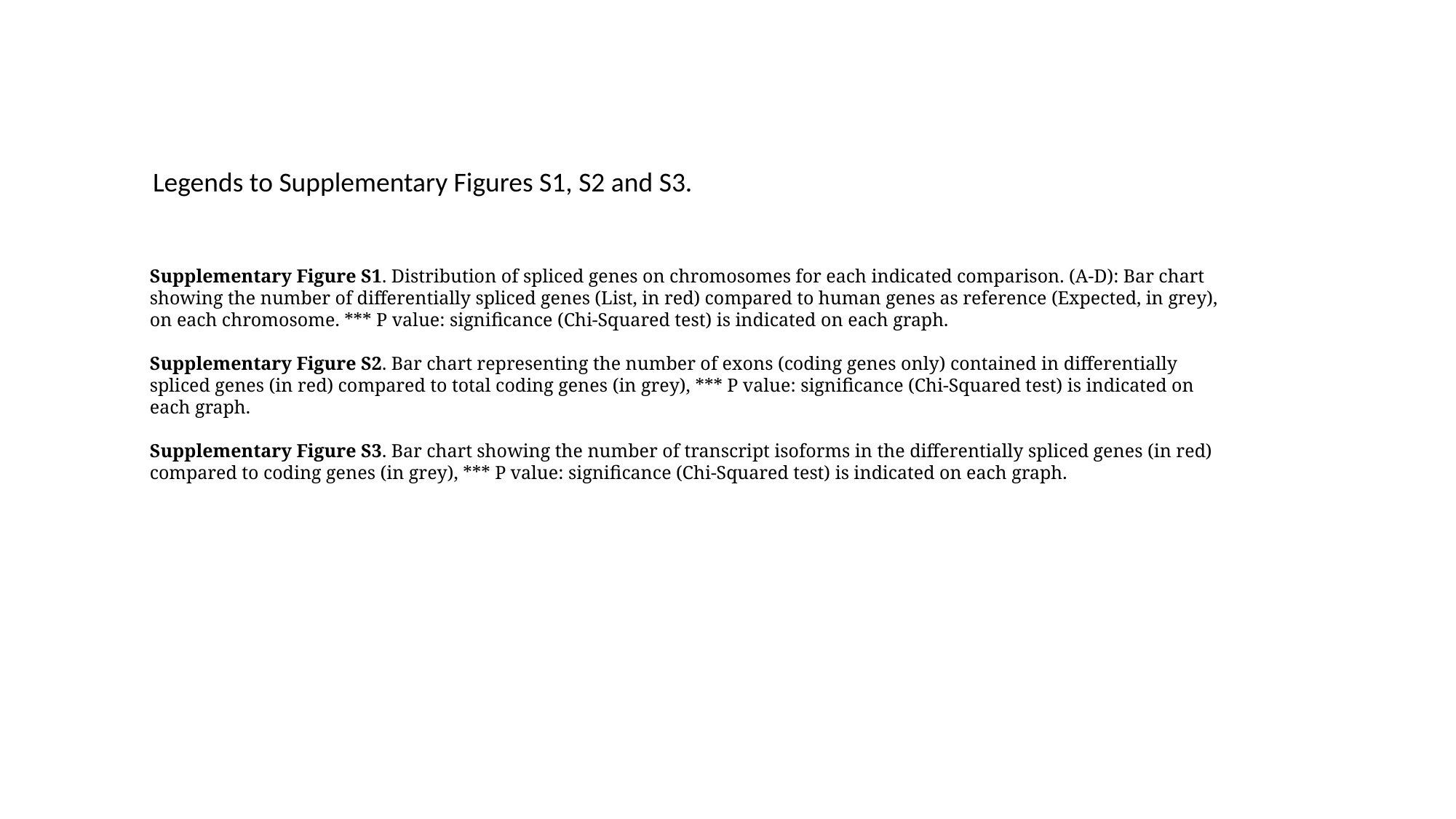

Legends to Supplementary Figures S1, S2 and S3.
Supplementary Figure S1. Distribution of spliced genes on chromosomes for each indicated comparison. (A-D): Bar chart showing the number of differentially spliced genes (List, in red) compared to human genes as reference (Expected, in grey), on each chromosome. *** P value: significance (Chi-Squared test) is indicated on each graph.
Supplementary Figure S2. Bar chart representing the number of exons (coding genes only) contained in differentially spliced genes (in red) compared to total coding genes (in grey), *** P value: significance (Chi-Squared test) is indicated on each graph.
Supplementary Figure S3. Bar chart showing the number of transcript isoforms in the differentially spliced genes (in red) compared to coding genes (in grey), *** P value: significance (Chi-Squared test) is indicated on each graph.
