# Supplementary material for: Impact of IDH Mutations, the 1p/19q Co-Deletion and the G-CIMP Status on Alternative Splicing in Diffuse Gliomas
Source: Int J Mol Sci. 2023 Jun 6;24(12):9825. doi: 10.3390/ijms24129825 (PMC10297931; doi:10.3390/ijms24129825)
Supplement: Supplementary file 1 [file ijms-24-09825-s001.zip › Zhang et al - Supp Fig 4&5 - IJMB-1June2023.pptx]

## Slide 1
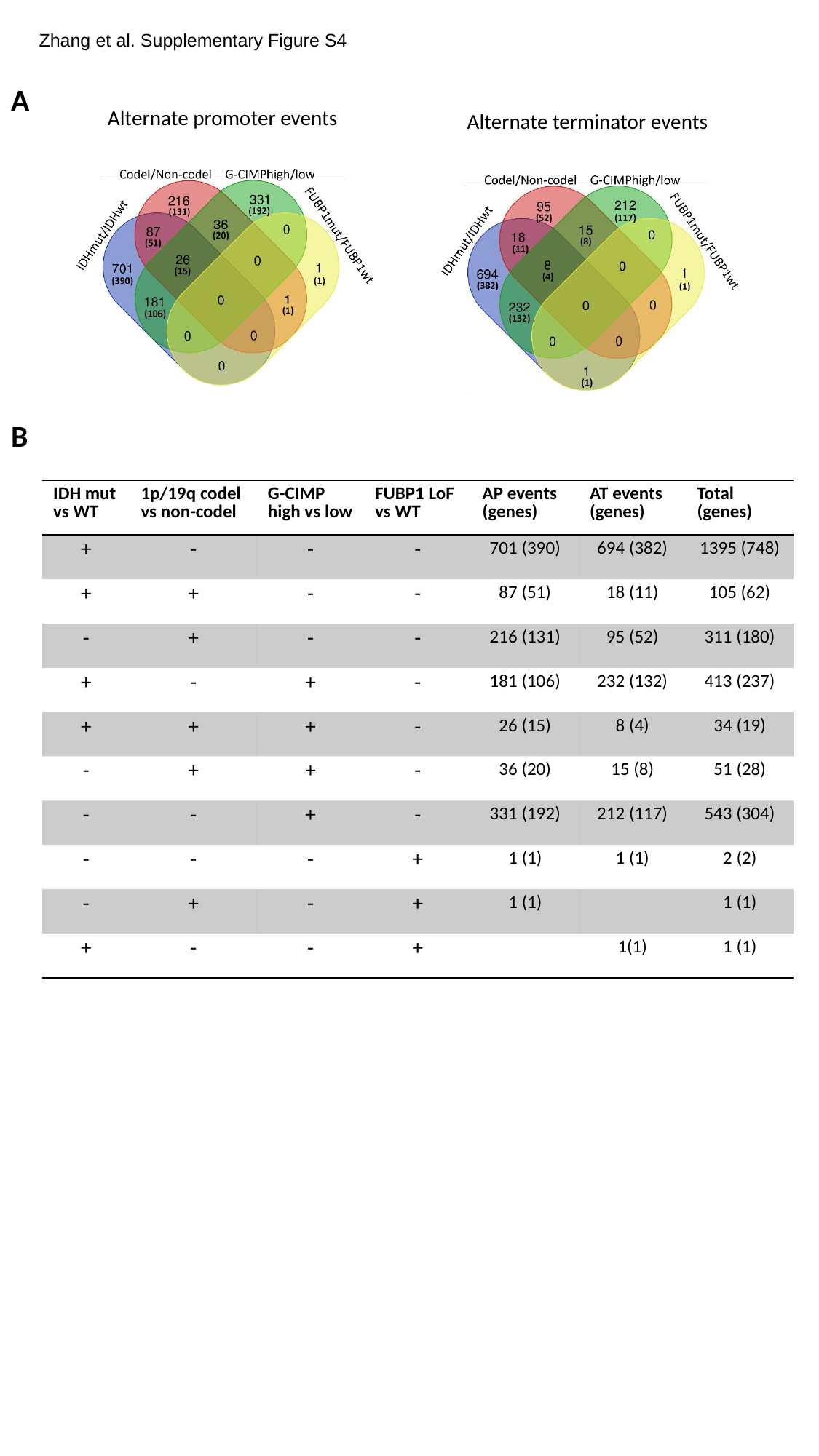

Zhang et al. Supplementary Figure S4
A
Alternate promoter events
Alternate terminator events
B
| IDH mut vs WT | 1p/19q codel vs non-codel | G-CIMP high vs low | FUBP1 LoF vs WT | AP events (genes) | AT events (genes) | Total (genes) |
| --- | --- | --- | --- | --- | --- | --- |
| + | - | - | - | 701 (390) | 694 (382) | 1395 (748) |
| + | + | - | - | 87 (51) | 18 (11) | 105 (62) |
| - | + | - | - | 216 (131) | 95 (52) | 311 (180) |
| + | - | + | - | 181 (106) | 232 (132) | 413 (237) |
| + | + | + | - | 26 (15) | 8 (4) | 34 (19) |
| - | + | + | - | 36 (20) | 15 (8) | 51 (28) |
| - | - | + | - | 331 (192) | 212 (117) | 543 (304) |
| - | - | - | + | 1 (1) | 1 (1) | 2 (2) |
| - | + | - | + | 1 (1) | | 1 (1) |
| + | - | - | + | | 1(1) | 1 (1) |

## Slide 2
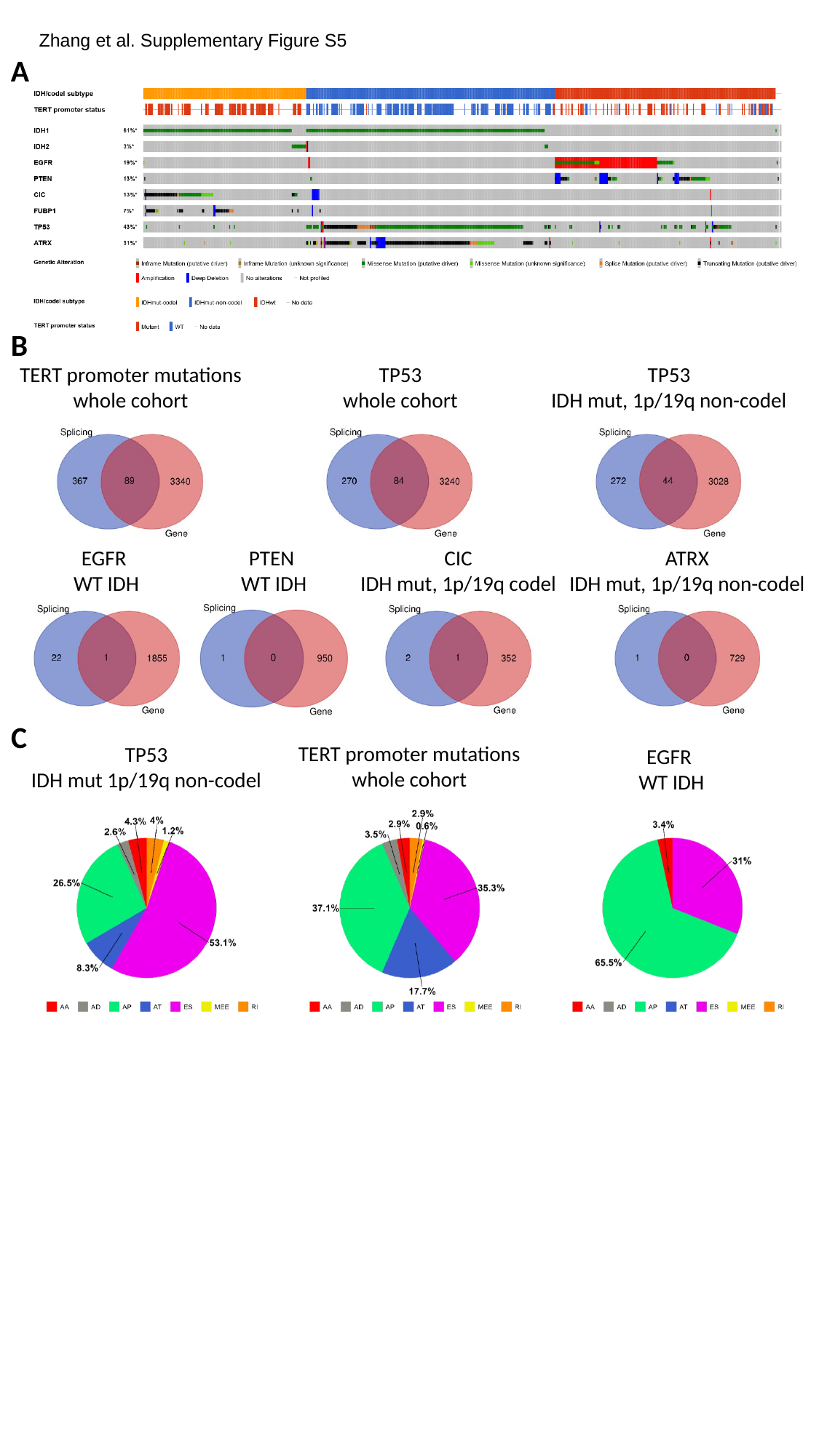

Zhang et al. Supplementary Figure S5
A
B
TERT promoter mutations
whole cohort
TP53
whole cohort
TP53
IDH mut, 1p/19q non-codel
EGFR
WT IDH
PTEN
WT IDH
CIC
IDH mut, 1p/19q codel
ATRX
IDH mut, 1p/19q non-codel
C
TERT promoter mutations
whole cohort
TP53
IDH mut 1p/19q non-codel
EGFR
WT IDH

## Slide 3
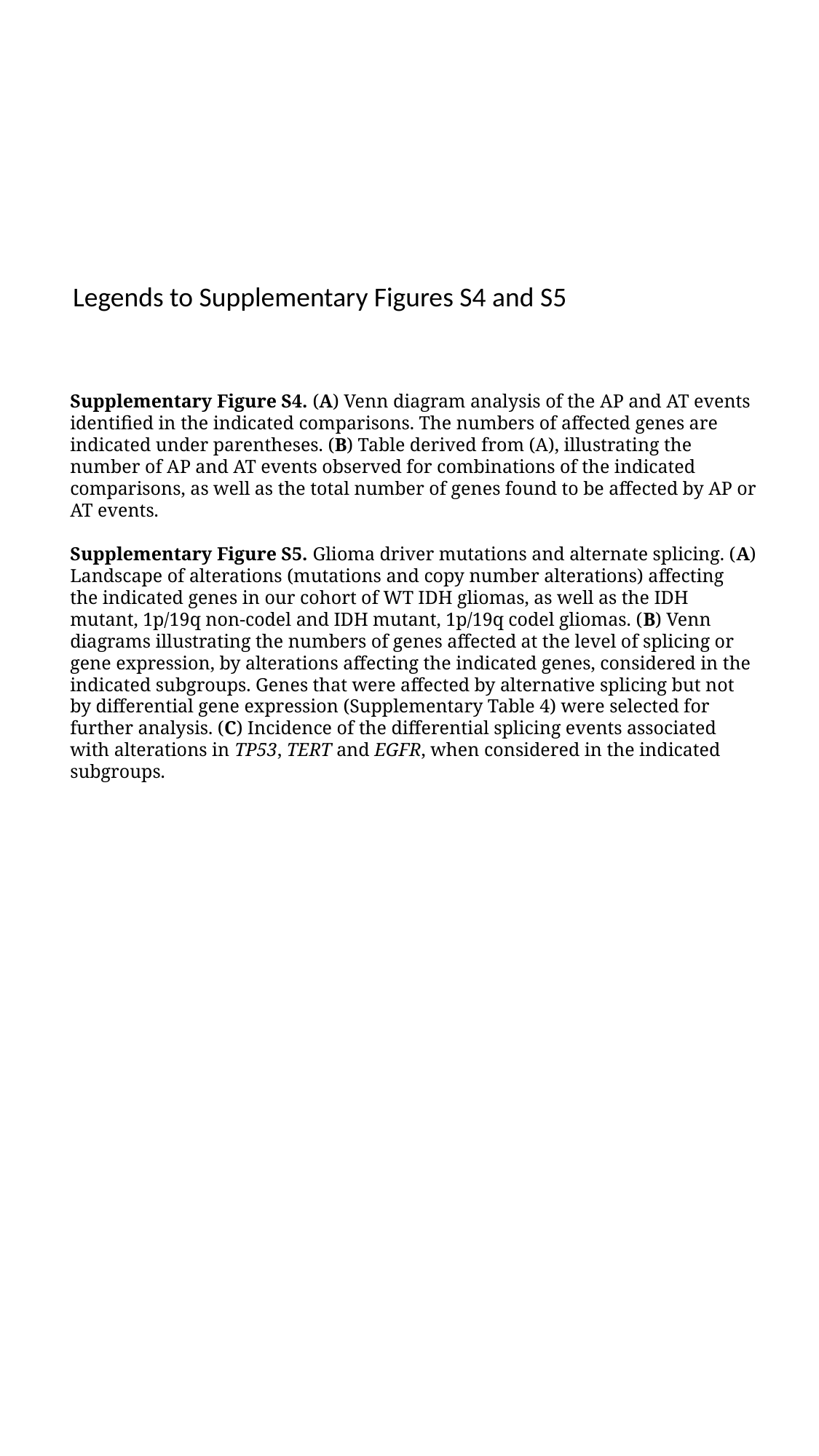

Legends to Supplementary Figures S4 and S5
Supplementary Figure S4. (A) Venn diagram analysis of the AP and AT events identified in the indicated comparisons. The numbers of affected genes are indicated under parentheses. (B) Table derived from (A), illustrating the number of AP and AT events observed for combinations of the indicated comparisons, as well as the total number of genes found to be affected by AP or AT events.
Supplementary Figure S5. Glioma driver mutations and alternate splicing. (A) Landscape of alterations (mutations and copy number alterations) affecting the indicated genes in our cohort of WT IDH gliomas, as well as the IDH mutant, 1p/19q non-codel and IDH mutant, 1p/19q codel gliomas. (B) Venn diagrams illustrating the numbers of genes affected at the level of splicing or gene expression, by alterations affecting the indicated genes, considered in the indicated subgroups. Genes that were affected by alternative splicing but not by differential gene expression (Supplementary Table 4) were selected for further analysis. (C) Incidence of the differential splicing events associated with alterations in TP53, TERT and EGFR, when considered in the indicated subgroups.
